# Dendritic Cell-Based Vaccination in Cancer: Therapeutic Implications Emerging from Murine Models

**DOI:** 10.3389/fimmu.2015.00243

**Published:** 2015-05-20

**Authors:** Soledad Mac Keon, María Sol Ruiz, Silvina Gazzaniga, Rosa Wainstok

**Affiliations:** ^1^Laboratorio de Cancerología, Fundación Instituto Leloir, Instituto de Investigaciones Bioquímicas de Buenos Aires IIBBA-CONICET, Buenos Aires, Argentina; ^2^Centro de Investigaciones Oncológicas, Fundación para la Investigación, Docencia y Prevención del Cáncer (FUCA), Buenos Aires, Argentina; ^3^Laboratorio de Biología Tumoral, Departamento de Química Biológica IQUIBICEN-CONICET, Facultad de Ciencias Exactas y Naturales, Universidad de Buenos Aires, Buenos Aires, Argentina

**Keywords:** cancer immunotherapy, dendritic cell-based vaccines, dendritic cells, dendritic cell maturation, dendritic cell subsets, adjuvants

## Abstract

Dendritic cells (DCs) play a pivotal role in the orchestration of immune responses, and are thus key targets in cancer vaccine design. Since the 2010 FDA approval of the first cancer DC-based vaccine (*Sipuleucel-T*), there has been a surge of interest in exploiting these cells as a therapeutic option for the treatment of tumors of diverse origin. In spite of the encouraging results obtained in the clinic, many elements of DC-based vaccination strategies need to be optimized. In this context, the use of experimental cancer models can help direct efforts toward an effective vaccine design. This paper reviews recent findings in murine models regarding the antitumoral mechanisms of DC-based vaccination, covering issues related to antigen sources, the use of adjuvants and maturing agents, and the role of DC subsets and their interaction in the initiation of antitumoral immune responses. The summary of such diverse aspects will highlight advantages and drawbacks in the use of murine models, and contribute to the design of successful DC-based translational approaches for cancer treatment.

## Introduction

Dendritic cells (DCs) form a network of antigen-presenting cells (APCs) that shape immune responses by linking innate and adaptive immunity. Their ability to induce or suppress the proliferation, activation, and differentiation of specific T-cell subsets renders them an attractive target for cancer immunotherapies.

Tumoral antigens are mostly self-proteins (1). As a result, cancer is associated with T-cell deletion and a defective T-cell memory repertoire, which includes anergic CD8^+^ T cells (hyporesponsive state upon low co-stimulation or high co-inhibitory stimulation) and exhausted CD8^+^ T cells (decreased cytokine expression and effector function upon chronic activation), as recently reviewed (2, 3). However, T cells recognizing tumor-specific antigens and tumor-associated antigens (TAAs) that can be targeted by vaccination have been described in the context of cancer (1, 4, 5). Therefore, therapeutic vaccines should be able to prime naïve T cells, but most importantly, induce the transition of existing memory T cells from non-protective to potent effector CD8^+^ T cells (6). DCs are considered the most efficient APCs (7). In order to instruct naïve T cells into the required functional profile, DCs must present tumor antigens via MHC class I and II molecules (signal 1), express co-stimulatory ligands (signal 2), and inflammatory mediators (signal 3) such as IL-12 or type I interferons (IFNs) (8, 9). Recent works have highlighted the importance of the innate immune system in the tumor microenvironment. This is the site where initial antigen sampling and activation of APCs take place, including different subsets of DCs. It was shown that a T-cell inflamed tumor microenvironment phenotype is associated with clinical responses to therapeutic cancer vaccines (10). Therefore, a complete description of the functional specialization and tissue distribution of DCs deserves special attention in tumor models.

For over 20 years, clinical trials have been performed using DC-based vaccines, and they have proven to be feasible, safe, and to elicit immunological responses (11). Recent reviews that have outlined the clinical effects of DC-based vaccines highlight the discrepancy observed between the immunonogenicity and the therapeutic efficacy in terms of inducing tumor rejection (6, 12, 13). This clinical challenge emphasizes the importance of employing adequate murine models in pre-clinical research. The availability of different genetic backgrounds allows researchers to gain insight into the function and relevance of specific DC subsets, track vaccine cells once injected, evaluate antigen-specific T-cell proliferation and activation, and work with human tumor cells in humanized mouse models. In this review, the knowledge gained through murine models will be summarized, with the aim of helping in the design of efficient DC-based vaccines that can be rapidly translated to human clinical trials.

## Murine Models in Cancer Research

Murine models have been an invaluable tool in the study of DCs and cancer immunotherapy. It was in mouse lymph nodes that DCs were first identified by Steinman et al. as a novel cell type with distinct morphological features (14). Regarding tumor immunology, the first evidence of the immune system’s ability to recognize tumoral antigens and reject tumors was obtained using mice with implanted or chemically induced tumors (15) or infected with oncogenic viruses (16). Since then, much has been learned in this field with the aid of wild type and genetically engineered mice (GEM) strains. The approaches used to generate these GEMs and some of their limitations have been reviewed in Ref. (17).

Murine cell lines have been established for a variety of tumor cell types in many genetic backgrounds. The B16 melanoma cell line and its derived sublines B16-F1 and B16-F10 (18) have been used extensively as syngeneic transplants into C57BL/6 mice. B16 cells are poorly immunogenic, and express low levels of MHC class I molecules (19). Therefore, immunotherapeutic strategies that generate tumoral protection in other tumor models do not work as efficiently with B16 tumors (20). For this reason, it is a good model to evaluate the strength of antitumoral immunotherapies.

Although it has proved very useful for the evaluation of anti-melanoma immunotherapies, this model has several features that do not entirely reflect the human disease. Principally, the frequently observed BRAF^V600E^ mutation in human melanomas (21) is absent from B16, B16-F1, and B16-F10 cells, which show a distinct pattern of genetic alterations (22). In this context, GEMs with conditional expression of the *BRAF*^V600E^mutation or other mutations frequently observed in human melanomas have been developed. Moreover, human melanomas may take years to become clinically evident, and dormancy periods between primary tumor resection and metastatic disease offer a time-window for therapeutic interventions aiming to prevent future recurrence in high-risk patients. By contrast, all B16 cell lines cause rapid *in vivo* growth, leading to measurable tumors within 15–20 days of subcutaneous (s.c.) transplantation, and compromising the study of therapeutic approaches. Their rapid s.c. growth does not give cancer cells time to progress into metastatic disease without previous resection of the primary tumor, poorly recapitulating human clinical stages. To study metastatic disease, the more aggressive B16-F10 cell line is usually injected directly intravenously (i.v.). However, stages such as detachment of tumor cells from the primary tumor mass and intravasation into nearby vessels are not represented. GEMs with *PTEN* deficiency and melanocyte-specific induced *BRAF*^V600E^ mutation develop malignant melanoma with posterior metastasis to lymph nodes and lung (23), more closely resembling human tumor progression. The main challenge to widespread implementation of GEM models is the technical difficulty and high cost of generating these strains. Recently, there have been *BRAF*^V600E^ cell lines isolated from transgenic *BRAF*^V600E^ mice and *BRAF*^V600E^ *PTEN*^−/−^ mice (24). These cells recapitulate human *BRAF*^V600E^ melanoma *in vitro*, and can be transplanted into syngeneic mice for assessment of host-tumor interactions. Using knock-in technology, mutations can be targeted to the endogenous gene locus of interest, and so innate regulation of gene expression can be achieved. Some examples of GEMs that target oncogenes or tumor suppressor genes and that may be useful for assessing the efficacy of tumor vaccines are those that target KIT, TRP53, BRAF, KRAS, PIK3CA, and EGFR (25).

The ovalbumin (OVA)/OT-I and OT-II murine model have been used to study specific T-cell responses. Briefly, mice are immunized with the OVA antigen (whole protein or peptides), and then OVA-specific CD4^+^ or CD8^+^ T-cell restricted responses are assessed using T cells obtained from transgenic animals that recognize OVA epitopes on MHC class I molecules (CD8^+^ T OT-I cells) or MHC class II molecules (CD4^+^ T OT-II cells). Though this model has proven very informative in immunology, it diverges critically from the cancer setting in that OVA is an exogenous antigen, and so OT-I and OT-II cells have not been subjected to negative selection or peripheral tolerance. This model can provide information about naïve T-cell distribution and activation but does not reflect the behavior of human tumor-specific clones; so, the results obtained should be evaluated carefully. As will be discussed later, the success of a particular immunotherapeutic strategy may vary depending on whether the antigen is a self-peptide or an exogenous peptide.

An intermediate model between human cell culture and mouse cancer models is the transplantation of human surgical specimens or established cell lines (xenografts) into immunocompromised mice. These have been used successfully to study human cancer cells in an environment that better reflects tumoral complexity and architecture (26). But when these models are used, it is not possible to assess the patient’s immune response to the tumor. This can be solved by the use of “humanized” mice, severely immunodeficient mice with mutations in the IL-2 receptor common γ-chain locus, which can be engrafted with human peripheral blood mononuclear cells (PBMCs) or human stem cells (HSCs) (27). The IL-2 receptor γ-chain is necessary for the binding and signaling several cytokines, and for NK development (28, 29), resulting in the absence of NK cells and improving human cell engraftment and the analysis of antitumoral responses (30). In the NOD-*scid Il2R*γ^−/−^strain, multilineage engraftment including human CD14^+^ cells, NK cells, and plasmacytoid BDCA-2^+^ DCs can be observed after HSC transplantation. The drawback is that these mice have severely hypoplastic peripheral lymph nodes, associated with impaired antibody class-switch and affinity maturation (31). They also have proportionally low numbers of human myeloid CD11c^+^CD86^+^ DCs, which fail to produce IL12p40 or IFN-γ after CD40 stimulation (30). It is thus difficult to evaluate DC migration to secondary lymphoid organs and their interaction with T cells to trigger adaptive responses. Nonetheless, T-cell responses *in vivo* using donor-matched DCs have been successfully assessed (32, 33). To facilitate the engraftment of different cell types, GEMs expressing human cytokines (34) or protocols administering such recombinant proteins have been developed, and are reviewed by Drake et al. (35).

Differences between the murine model and the human disease may partially account for the lower efficiency observed in human clinical trials. Hopefully, new models have been designed that better recapitulate human disease or that allow studying immunotherapies utilizing the patient’s own tumor and immune cells. Therefore, researchers should take special care selecting the model that best fits their objectives. The recommended applications and considerations for choosing a murine model for DC-based vaccination in cancer have been summarized in Table 1.

**Table 1:** **Advice for choosing murine models for DC-based tumor immunotherapy**.

Murine model	Applications	Disadvantages
Tumor cell lines transplanted into singeneic strains	Evaluate *in vitro* DC maturation, Ag presentation, and/or lymphocyte activation (36, 37)	Different pattern of genetic alterations than human tumors (22) [transgenic cell lines or cell lines isolated from GEMs, which carry human genetic alterations may be used (24)]
	DC activation *in vivo* and migration to draining lymph nodes (38–41)	
	DC targeting *in vivo* with receptor-specific antibodies (42, 43)	
	Evaluate tumor growth and response to treatment *in vivo*; dissemination after i.v. injection (44–46)	
	T cell activation *in vivo*, profile (effector or memory) of T cells (47–50)	
	Assess antibody production	
		Variable immunogenicity among cell lines
		Variable responses depending on the genetic background of the recipient strain. When choosing one model, there is a biased immune response (genetically identical hosts)
		Rapid *in vivo* growth which does not recapitulate human clinical tumoral progression, difficult to assess therapeutic approaches [modifications in tumoral challenge sites have been reported that allow the study of primary tumors and posterior dissemination to draining lymph nodes (51) or metastatic dissemination prior to local growth (52)]
GEMs	Mice are immunocompetent; so, immune responses can be studied	Technical difficulty and high cost
	Human genetic alterations can be induced in the tissue of origin (23)	Tumor development is slow and variable (23, 54)
	Tumoral protection can be assessed using a model that recapitulates human clinical stages, including variability in tumor phenotype. Appropriate to study therapeutic approaches (53)	
Xenografts in immunodeficient strains	Study human cancer cells in an environment that better reflects tumoral complexity and architecture (26)	Immune protection *in vivo* cannot be correctly assessed [human immune cells can be transplanted, but there is short-term persistence (55)]
		Human tumor stroma and lymphocytes are lost in the initial passages (26)
		Stromal, endothelial, and residual immune cells are from murine origin (56)
		Selective pressures induce clonal expansion of an original polyclonal tumor (57)
		Low engraftment of human tumors and immune cells
Xenografts in NOD-SCID IL2RY^−/−^humanized mice	Assess the patient’s immune response to the tumor	Hypoplastic peripheral lymph nodes (impaired antibody class switch and affinity maturation) (31)
	Study of human tumor-stromal interactions (human tumor microenvironment)	
	Test therapeutic efficacy of vaccines (32, 33, 59)	
	Study human DC subsets *in vivo* (60)	
		Graft-versus-host disease generated by human T cells reacting to murine leukocyte antigen molecules. NOD-SCID IL2Ry^−/−^strains lacking MHC-I or MHC-II have recently been developed (58)
		After engraftment, low numbers of human myeloid CD11c^+^CD86^+^ DCs, which fail to produce IL12p40 or IFN-γ after CD40 stimulation (30)
		To facilitate the engraftment of different immune cells, GEMs expressing human cytokines (34)or protocols administering such recombinant proteins have been developed (35)

## Lessons Learned from Murine Models

### Characterizing DC subsets

Recent reviews have described at length the ontogeny, phenotype, and transcriptional profile of the heterogeneous population collectively named DCs (61–63). This network relies on the differential expression of a group of transcription factors that determine the specification of the different subsets of DCs (64). Steady-state DCs can be classified into two groups: plasmacytoid DCs (pDCs) and classical/conventional DCs (cDCs). Two further subsets of cDCs can be distinguished in lymphoid tissues: CD8^+^ and CD11b^+^cDCs, while in non-lymphoid tissues, cDCs are classified into CD11b^−^CD103^+^ and CD11b^+^CD103^−^. Langerhans cells (LCs) represent an additional population of DCs that reside in the epidermis, although they can be found at draining lymph nodes both in the steady state and after an inflammatory stimulus. Lastly, during an inflammatory response, monocyte-derived DCs (MoDCs) are induced and recruited to the sites where the response was initiated, and migratory DCs can be found in draining lymph nodes.

**Figure 1:**
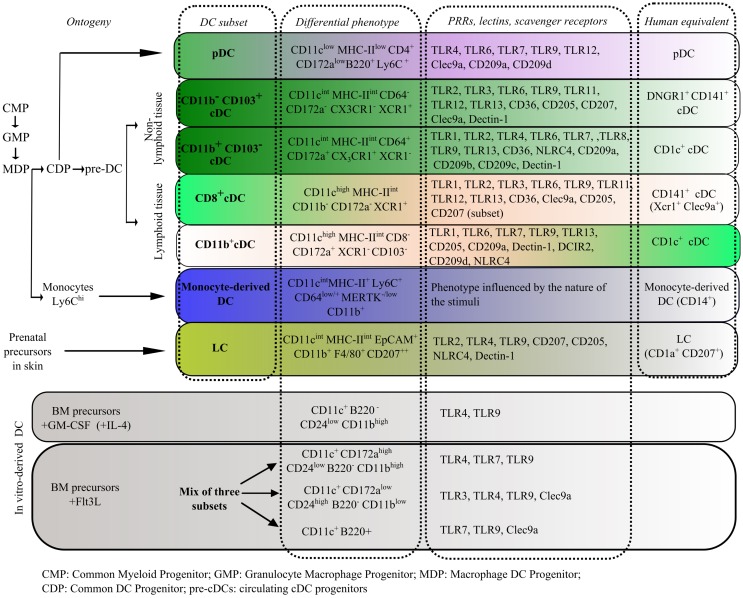
**Description of ontogeny, phenotype, and patterns of PRR expression in murine DC subsets in the steady state**. Comparison to the equivalent human subsets and to murine *in vitro*-derived DCs is provided (61, 62, 66–77).

Deeper insights at the molecular level have improved the distinction of DCs from other immune population, such as macrophages, by providing a list of transcripts that define a “core cDC signature.” This signature includes the chemokine receptor CCR7, the transcriptional regulator Zbtb46, the Flt3L receptor, and Kit (63). In coming years, transcriptional profiling should be a helpful tool in the difficult task of assigning specific functions to different DC population. So far, functional studies have shown that each subset has particular abilities regarding antigen processing, response to environmental signals, and the induction of naïve T cells into effector lymphocytes (65). The response to environmental signals is mediated by the expression of a set of innate pattern recognition receptors (PRRs) that can bind conserved antigen determinants of virtually all classes of pathogens, which are called pathogen-associated molecular patterns (PAMPs), and also recognize endogenous signals released during a stress or damage response (damage-associated molecular patterns, DAMPs). The pattern of expression of PRRs, scavenger, and lectin receptors on different DC subsets is of great importance to predict their potential activation in different physiological contexts, including the tumor microenvironment. Some of the most relevant phenotypic markers, PRRs, and precursors to each subset are listed in Figure 1. There are controversies regarding the involvement of particular DC subsets in tolerogenic responses to tumors. This section will focus on evidence regarding the observed contributions of specific DC subsets in immune responses elicited by DC-based vaccines in cancer.

#### Plasmacytoid DCs

Plasmacytoid DCs are found mainly in the blood and lymphoid tissues. Their unique properties and phenotype separate them from cDCs (Figure 1). Upon encounter with foreign nucleic acids, pDCs produce massive amounts of type I IFNs, which can activate both the innate and the adaptive arms of the immune system. Despite the large body of knowledge acquired about pDC biology, to date, their suitability for DC immunotherapy is only recently being explored. In support of an immunogenic pDC role, imiquimod (TLR7 agonist)-mediated regression of melanoma tumors was shown to be dependent on TLR7 expression on pDCs; neither mice lacking TLR7 nor pDC-depleted mice responded to imiquimod therapy and both saw a reduction in apoptosis within treated tumors (78). Other works have shown that the cross-presentation pathway is under the control of TLR activation (79). Moreover, results obtained from *in vitro* killing assays suggest that pDCs may exert direct cytotoxic effects on tumor cells by secreting soluble factors in response to imiquimod treatment (78). Recent works have highlighted previously unknown functions of pDCs in antitumor immunity. In work by Guery et al., antigen presentation by pDCs was required for efficient antigen-specific Th17 responses. Mice that were immunized with OVA plus CpG-B (synthetic TLR9 ligand) and possessed pDCs lacking MHC class II molecules showed reduced numbers of IL-17-secreting OT-II cells, exhibited a significant increase in tumor growth, and showed a reduction in the recruitment of tumor-specific cytotoxic T lymphocytes (CTLs) into tumors (80). In a different model, the injection of CpG-activated pDCs after a tumor challenge led to a significant delay in the growth of B16 melanoma and MCA205 sarcoma tumors, and induced the recruitment of antigen-specific CD8^+^ T cells and activated NK cells into tumors by a mechanism dependent on endogenous cDCs and NK cells. In this work, CpG-activated pDCs were able to stimulate NK cell cytolytic function by secretion of type I IFN (81). This latter works highlight the potential of activated pDCs to activate NK cells and orchestrate a cytotoxic immune response against tumoral antigens.

On the other hand, several evidences in the human setting support an immunosuppressive role for pDCs in the tumor microenvironment: tumor-associated pDCs (TApDC) correlated with unfavorable prognosis in patients with breast cancer (82), where they were found to colocalize and correlate with tumor-associated regulatory T cells (Tregs) (83); ovarian carcinoma ascites-derived pDCs induced IL-10-producing Tregs *in vitro* (84); tolerogenic pDCs were found infiltrating prostate tumors (85), and together with inducible costimulator (ICOS)-expressing FoxP3^+^Tregs, pDCs were strong predictors for disease progression in patients with ovarian cancer (86). In mouse models, fewer works have studied the mechanisms of pDCs-induced immunosuppression. Watkins et al. used a transgenic adenocarcinoma of mouse prostate to study tumor infiltrating DCs, and found a predominant pDC population able to induce tolerance in antigen-specific T cells. Furthermore, they studied the role of transcription factor Foxo3 in mediating TApDC-induced suppression (85). In accordance, the depletion of pDCs in mice bearing CpG-treated Lewis lung carcinoma (LLC) tumors correlated with lower numbers of intratumoral Tregs, an increase in the recruitment of mature cDCs, and the arrest of tumor growth (87). Delayed tumor growth was also observed after pDC depletion in an orthotopic mammary tumor model (88). However, in both works, pDC depletion was induced by treatment with the m-927 antibody, which recognizes the antigen BST2. Although this antigen is mostly restricted to pDCs in the steady state, it is upregulated in several cell types following activation. In fact, pDCs and plasma cells are indistinguishable based on BST2 expression after stimulation (89). Therefore, conclusions made about pDC-depletion experiments should take this confounding factor into account. Additional evidence includes indoleamine 2,3-dioxygenase (IDO)-expressing pDCs that were found at draining lymph nodes of tumor-bearing mice. These IDO-pDCs showed ability to activate Tregs *in vivo* (90). However, pDCs were defined in this system as CD11c^+^B220^+^CD19^+^ cells, which it is still not clear if it represents a rare subset of pDCs that express CD19, or a distinctive B-lymphoid cell type with T-cell regulatory features and phenotypic features of both B cells and DCs (91, 92).

Of main importance for translational research is the possible modulation of murine and human pDC immunosuppressive activity into an effective protective phenotype. *In vivo* intratumoral administration of TLR7 ligands led to ICOS^+^ TApDC activation and tumor regression in an orthotopic mammary tumor model (88). In human breast cancer, *in vitro* neutralization of ICOS molecule blocked pDC-induced Treg expansion and reduced IL-10 secretion by memory TA-CD4^+^ T cells (93).

#### Langerhans Cells

Epidermal LCs were originally described as prototype DCs because of their ability to capture antigens in the skin and migrate to draining lymph nodes in response to foreign stimuli. Their strategic location makes them attractive targets for epicutaneous immunization or i.d. vaccination. However, recent discoveries about their ontogeny and functional redundancy have set them apart from other DCs. As opposed to their dermal counterparts, LCs can originate from radio-resistant precursors present in the skin, and their development is independent of Flt3L and Flt3, and dependent on TGF-β1 (94, 95). Since finding that other DCs population express the classical LC marker, langerin (CD207) (96, 97), functions that used to be attributed to LCs are being reevaluated and assigned to dermal CD207^+^ DCs (65, 98–100). Although CD207^+^ cells can mediate efficient antigen presentation (100–103), recent works have shown that targeting antigens to LCs via CD207 induces antigen-specific cross-tolerance, while targeting antigens to dermal CD207^+^ DCs elicits a cytotoxic response and long-lived immunological memory. Surprisingly, the tolerogenic effect mediated by LCs occurred only in the presence of adjuvants, and not in the steady state. It is worth noting that authors observed an initial proliferation response, regardless of the adjuvant used, and only detected a difference between treatments by analyzing the surviving OT-I CD8 T cells, which displayed a phenotype of central memory T cells, at 8 weeks after immunization (47). Such deep analysis of immune responses should be encouraged in all experimental approaches when possible.

#### Lymphoid Tissue CD8^+^ and Non-Lymphoid Tissue CD11b^−^ CD103^+^cDCs

CD8^+^ and CD103^+^cDCs share many phenotypic and functional features, including the expression of XCR1 shared by human CD141^+^cDCs (Figure 1). They also display similar transcriptional profiles (63). Given their superior ability to cross-present antigens to CD8^+^ T cells, they are especially suitable as inducers of antitumoral responses (66). Mice with defective Batf3 (transcription factor also known as Jun dimerization protein p21SNFT) lack splenic CD11c^high^CD8^+^CD205^+^ cells and show reduced numbers of CD11b^low/−^CD103^+^ dermal DCs in skin-draining lymph nodes. Unlike their wild-type controls, Batf3^−/−^ mice failed to reject syngeneic fibrosarcomas, did not develop tumor-specific CTLs, and showed reduced numbers of tumor-infiltrating CD8^+^ T cells (104). A similar pattern was observed in a model of B16. SIY melanoma, in which CD8^+^cDCs were critical for the spontaneous priming of tumor-specific CD8^+^ T cells (105). In this case, the mechanism was shown to be dependent on type I IFN signaling in the CD8^+^cDCs lineage (105, 106). However, the identity of the specific DC subset of cells producing type I IFNs in response to tumor growth remains to be elucidated, suggesting a collaborative process for induction of effective antitumor T cell priming. In support of these observations, CD8^+^cDCs pulsed with tumor lysates were able to reduce tumor growth in fibrosarcoma-bearing mice (107).

Lymphoid tissue CD8^+^cDC subset is a heterogeneous population. CD207^+^CD8^+^cDCs were the only splenic DCs capable of cross-presenting a systemic soluble protein antigen *in vitro* and *in vivo* to CD8^+^ T cells, though their role in antigen presentation via MHC class II molecules to CD4^+^ T cells was limited (108). Furthermore, CD207^+^CD8^+^cDCs were the main producers of IL-12 after systemic injection of the iNKT cell ligand α-galactosylceramide (α-GalCer), and contributed to the initial burst of antigen-specific CD8^+^ T cell proliferation after i.v. injection of the OVA protein plus α-GalCer (108). Accordingly, co-delivery of the protein antigen and α-GalCer to CD8^+^cDCs by nanoparticles coated with anti-CD205 antibodies elicited potent antitumor responses in a preventive scheme and delayed tumor growth in a therapeutic setting, suggesting that this strategy optimizes iNKT cell-mediated immune response (109). Furthermore, a recent work has identified CD205^+^ CD8^+^cDCs as the key APCs for multiple forms of α-GalCer (110). Of translational relevance, these authors showed how the expression of costimulatory and coinhibitory molecules on the surface of CD8^+^cDCs, such as CD70, Rae-1, PD-L1, and PD-L2, was modulated in response to different iNKT cell agonists. Future experiments on human cells should take into account that, in contrast to the murine system, there are five human isoforms of CD1, with differential ability to bind and present lipidic antigens to T cells (111).

In non-lymphoid tissue, cDCs represent 1–5% of the cells. In the case of the mouse intestine, CD11b^−^CD103^+^cDCs are enriched in Peyers’ patches and their putative human counterparts are DNGR-1^+^CD141^+^CD11b^−^DCs (112). CD11b^−^CD103^+^CD8α^+^ have been shown to migrate from intestine to mesenteric lymph nodes, and to be able to induce OVA-specific CD8^+^ T-cell proliferation *in vitro* (113). In the lung, CD11b^−^CD103^+^ were the only DC subset able to acquire and transport apoptotic cells to draining lymph nodes, and cross-present apoptotic cell-associated antigen to CD8^+^ T cells (114). Their strategic location makes them suitable targets for mucosal immunization. However, their relevance in tumor models remains poorly understood. In fact, works on mucosal immunization have not addressed the role of different DCs subsets in the outcome of vaccination. In a murine model of human papillomavirus-associated genital cancer, s.c. vaccination route was shown to be superior to mucosal (intranasal and intravaginal) immunization route for inducing regression of established genital tumors (115). By contrast, in a model of orthotopic head and neck cancer, intranasal immunization provided better prophylactic and therapeutic efficacy than intramuscular vaccination (116). In a model of murine spontaneous adenomatous polyps, intrarectal immunization decreased tumor formation and prevented progression to invasive colorectal cancers, inducing potent local cellular and humoral responses (117). Elucidating the role of specific DCs subsets during mucosal immunization in diverse tumor models is required in order to exploit their potential, and represents an exciting field of research.

Much of the research on tumor immunology has focused on the process of antigen presentation at lymph nodes rather than local mechanisms that can occur at the tumor niche, which likely influence the function of tumor CTLs. A recent work by Broz et al. has elegantly dissected the composition of the myeloid tumor microenvironment across a broad range of tumors (118). They found a rare population of immunostimulatory cells at the tumor, corresponding to CD103^+^cDCs that were capable of inducing TCR signaling in both naïve and previously activated OT-I CD8^+^ T cells in ectopic and spontaneous mouse tumor models. Furthermore, characterization of different immune population highlights the existence of functional diversity among intratumoral APCs. In a different approach, Woo et al. showed that host APCs in the tumor microenvironment can incorporate tumor-derived DNA, which is proposed to be involved in the activation of the STING pathway, leading to IFN-β production and priming of CD8^+^ T cells against tumor antigens *in vivo* (119). These findings highlight that triggering innate immune activation against tumors can be a suitable antitumoral strategy if the adequate APC population is targeted.

#### Classical CD11b^+^ CD8^−^cDCs

Among non-lymphoid tissue cDCs, the transcriptional profile of the CD11b^+^CD103^−^ subset reveals that the current classification based on phenotypic markers defines a heterogeneous population, with some cells belonging to the cDC lineage and others to the macrophage lineage (63). Furthermore, the phenotypic and functional features of this subset can vary depending on the tissue they reside. In the intestinal lamina propia, CD11b^+^ CD8^−^cDCs comprise two population: CD11b^+^ CD8^−^ CD103^+^ cDCs express CCR7 and represent the primary DCs subset for antigen sampling and migration to mesenteric lymph nodes, whereas the CD11b^+^ CD8^−^ CD103^−^ subset represents a non-migratory gut-resident population with slower turnover rates (120).

Classical CD11b^+^ CD8^−^cDCs most often predominate the lymphoid-resident cDCs population. They comprise a heterogeneous population of DCs that are preferentially involved in MHC class II-restricted antigen presentation to CD4^+^ helper T cells (66). At least two subsets can be distinguished in the spleen based on their dependence on Notch signaling for differentiation (121). CD11b^+^ CD8^−^cDCs in the spleen can be detected at the red pulp and marginal zone (66), and so they represent a potential target for i.v. immunization. The candidate vaccine CyaA-Tyr (detoxified adenylate cyclase carrying a HLA-A*02-restricted tyrosinase 369–377 CTL epitope), already being evaluated in clinical trials (study identifier: PC1O0VAC02), targets CD11b^+^ cells (122). Dadaglio et al. showed that i.v. immunization induced antigen-specific CTL responses in mice by a mechanism dependent on activation of the TLR4/TRIF pathway in cDCs. *In vivo* experiments showed that the vaccine binds mainly to the CD11b^+^ CD8^−^ DCs subset in the spleen, and induced DCs activation and maturation. Surprisingly, CyA-Tyr administration induced upregulation of CD86, CD40, and MHC class II molecules on both CD8^+^CD11b^−^ and CD8^−^CD11b^+^cDCs, probably by indirect and direct effects, respectively (123).

#### Monocyte-Derived DCs

This subset, also described as inflammatory DCs, refer to a population of DCs that can be found transiently in response to inflammatory stimuli. MoDCs are phenotypically defined as CD11c^int^CD11b^+^Ly6C^+^ cells (Figure 1); however, their distinction from macrophages has been challenging (124). Ly6C^high^ monocytes and early hematopoietic precursors upon TLR engagement can serve as precursors (125, 126). Their putative human equivalent has been recently described as CD11c^+^ MHC class II^+^ CD16^−^ CD1c^+^ cells, with dendritic morphology and robust T-cell stimulatory capacities. When cultured with allogeneic naïve CD4^+^ T cells, this population was capable of potent stimulation of Th17 responses (127).

Several evidences about the role of MoDCs in models of infection and inflammatory diseases have been recently reviewed (124). Ma et al. have recently highlighted the participation of MoDCs in immune recruitment to the tumor bed in response to chemotherapy. These cells were found infiltrating tumors after anthracycline-based chemotherapy in an ATP-dependent fashion, and efficiently presented tumor antigens to CD8^+^ T cells *in vitro* and *in vivo* (128). However, their relevance in diverse tumor models has been incompletely described, and further research on this subset may provide novel and useful data about their potential modulation for cancer immunotherapies.

#### *In Vitro* Bone Marrow-Derived DCs

Given the low number of DCs that can be obtained from primary culture of non-lymphoid and lymphoid tissue, the possibility of obtaining high numbers of DCs from murine progenitors *in vitro* has greatly contributed to the knowledge of DC biology. There are two main experimental approaches to obtain murine DCs, and they are both based on the culture of bone marrow cells with specific cytokines. However, they give rise to DC population differing in phenotype, morphology, functional properties, and resemblance to *in vivo* subsets (Figure 1) (67).

In the first approach, bone marrow precursors are cultured with GM-CSF alone, or with the addition of IL-4 (129). These cells display an immature phenotype (low expression of co-stimulatory and MHC class II molecules), express high levels of CD11b, and have a low migrating capacity to draining lymph nodes after s.c. injection (130). They can secrete IL-12p70, TNF-α, IL-10, RANTES, and CCL2 in response to TLR agonists, and confer protective immunity against tumor challenge in the presence of maturation agents such as CD40L or after co-culture with apoptotic/necrotic tumor cells (130, 131). The absence of IL-4 during *in vitro* culture leads to the presence of additional immune population, such as F4/80^+^ macrophages and Ly6G^+^ neutrophils (130, 132).

In the second approach, bone marrow precursors are cultured with Flt3L (133). This culture is comprised of at least three different DCs population that, according to their phenotype and pattern of TLR, chemokine receptor expression, and cytokine production, highly resemble three DCs subsets *in vivo*: B220^+^pDCs, CD24^high^CD11b^low^ DCs (equivalent to CD8^+^cDCs), and CD24^low^CD11b^high^ DCs (equivalent to CD8^−^ cDCs). In the absence of adjuvants, only the CD24^high^CD11b^low^ subset could efficiently cross-present antigens to CD8^+^ T cells (68). Flt3L-derived DCs can also secrete IL-12p70 and RANTES in response to TLR agonists, while the opposite was found regarding TNF-α, IL-10, and CCL2. Their ability to migrate to draining lymph nodes after s.c. injection was superior to GM-CSF/IL-4-derived DCs (67).

In contrast with the mouse system, *in vitro*-derived human DCs are usually obtained from peripheral blood monocytes cultured with GM-CSF and IL-4. These cultures render high numbers of cDCs. Other approaches include culturing CD34^+^ progenitor cells with GM-CSF and TNF-α, or with Flt3L and thrombopoietin (134), or expansion of cord blood Lin^−^ cells followed by a differentiation step with SCF, GM-CSF, IL-4, and Flt3L (135). These latter cultures allow the generation of CD141^+^ DC subset, equivalent to mouse CD8^+^ DCs, and XCR1^−^ DCs, equivalent to MoDCs (136). Recently, an improved culturing method has shown to support the development of all the three major types of DCs (CD1c^+^ cDCs, CD141^+^ cDCs, and pDCs) used a combination of mouse BM stromal cells and defined human cytokines (Flt3L, SCF, and GM-CSF). These cultures also produced granulocytes, monocytes, NK cells, and B cells, and they resemble their blood-derived counterparts as assessed by gene expression, surface phenotype, and cytokine production (137).

#### Antigen Transfer Between APCs and Interaction Between DC Subsets

A novel mechanism for the induction of specific CD8^+^ T cell responses upon vaccination with DCs is antigen transfer between *ex vivo*-loaded DCs and resident DCs. Recent papers in which selective DC subsets are ablated have allowed authors to identify key players in the induction of immune responses. Petersen et al. made use of transgenic mice in which CD207^+^ cells can be selectively depleted to show how resident CD207^+^ CD8α^+^ DCs are required for efficient induction of CD8^+^ T cells, but not of the CD4^+^ T cell counterpart, after the administration of OVA-loaded, bone marrow-derived DCs (108, 138). Antigen transfer has also been reported in endogenous DCs subsets such as LCs and dermal DCs (97, 139).

Using transgenic mice that express a specific MHC class II haplotype under the control of a CD11c promoter, Kleindienst et al. have shown that specific T cell responses following antigen-loaded DC vaccination are enhanced by endogenous DCs that express the correct restriction elements through an antigen transfer mechanism dependent on direct cell-to-cell interaction. In this setting, few injected DCs reached the draining lymph nodes, while most of them were retained at the injection site (140). This observation has been confirmed by several groups (38, 130), and it suggests a putative mechanism in which antigens are taken up *in situ* by endogenous DC.

Several other works support the notion that injected *in vitro*-derived DCs have a minor role in the direct priming of T cells *in vivo*. In a work by Yewdall et al., authors injected an OVA-pulsed, DC vaccine into mice with chimeric bone marrow that lacked the hematopoietic compartment necessary to present the OVA peptide. They showed that both i.v. and s.c. administration of OVA-loaded or OVA-expressing DCs required the transfer of antigens to host DCs for efficient CD8^+^ T-cell priming (141).

Given these observations, and the complexity of the DCs network, how can we exploit the potential synergy among different immune population? There is compelling evidence that antitumor responses may be enhanced by the interaction between different DCs subsets. In an original and novel approach, recruitment and activation of immune cells *in situ* were elicited by matrices implanted into s.c. pockets, containing CpG danger signals, GM-CSF, and tumor lysates. Vaccinated mice were challenged with live B16-F10 melanoma tumor cells at day 14 and showed significant protection from tumor-induced lethality (up to 90% survival rate). In this setting, the number of CD8^+^cDCs and pDCs at the vaccination site correlated strongly with the magnitude of protective antitumor immunity, and a significant expansion of antigen-specific CTLs was observed in the spleens of vaccinated mice. Furthermore, two doses of this vaccine elicited protection in 20–47% of mice in a therapeutic setting of established tumors (142). In a different approach, a peptide-pulsed CpG-activated pDC vaccine induced an antigen-specific CD8^+^ T cell response directly and also affected cDCs priming capacity, resulting in a synergic antitumor response. Although the detailed mechanism was not described, it required cell-to-cell contact (143).

Cross-dressing is a recently described mechanism for antigen presentation. It involves the transfer of intact peptide-MHC class I or II complexes from dead donor cells to DCs. DCs use these complexes to activate CD8^+^ or CD4^+^ T cells that are peptide-specific and restricted to the MHC genotype of the donor cells (144, 145). Although the role of cross-dressing in antitumor immunity requires further study, a recent work by Li et al. has shown that CD8α^+^ and CD103^+^ DCs induce proliferation of naïve and memory CD8^+^ T cells both by cross-dressing and cross-presentation of antigens (146). This novel mechanism could explain some of the observations of antigen transfer between host and injected DCs, or among different resident DCs subsets.

### Analysis of immunogenic tumoral antigens

#### Loading DCs with Tumoral Antigens

Tumor antigens originate from mutated or abnormally expressed endogenous proteins, including differentiation antigens, or are derived from viral proteins. The common strategy to load DCs with antigens is to use short peptides that, independent of the processing machinery, can bind directly to MHC molecules. However, the main drawback is the requirement of specific haplotypes to be identified for efficient antigen presentation. The utilization of longer peptides or whole proteins could overcome this problem and minimize tumor evasion.

Several tumor antigens are produced only by specific proteasomes (1, 147, 148). For example, cells exposed to IFN-γ express different proteasome subunits in addition to the standard counterparts and consequently a different spectrum of peptides with variable immunogenic potency (147). Mature DCs used for immunotherapy express immunoproteasomes and do not express constitutive proteasomes (149). Recent reports show that DC proteasome composition can be manipulated to increase antitumoral responses (150). In a phase I clinical trial, mature melanoma antigen-loaded human DCs were engineered to process antigens through constitutive proteasomes, proving to be superior inducers of antigen-specific T-cell immunity and clinical responses. As in this clinical trial, it becomes relevant to test the outcome of vaccination with functional assays to evaluate whether the induction of peptide-specific CD8^+^ T cell renders better tumor reactivity (151).

An obvious advantage in employing autologous tumor cells as antigen source for DCs is that they contain patient-specific mutated antigens that have not been subjected to central tolerance. It has been proven that mutated epitopes with single amino acid substitutions can provide tumor control (152). However, these formulations face significant difficulties, such as standardization, scarce autologous tumor cell samples, and technical issues regarding the establishment of successful cell cultures from those tumors. Consequently, the rationale for using allogeneic tumor cell lines instead of autologous tumor cells is that they share several common antigens that could serve as targets, generating comparable responses (153, 154).

Recurrence or relapse after therapies could be due to cancer stem cells being partially or totally untargeted by vaccination. Therefore, one novel and interesting approach is the use of isolated cancer stem cells from the tumor bulk as the source of antigens (155, 156). Mice vaccinated with DCs pulsed with cancer stem cell lysates significantly inhibited tumor growth and displayed higher amounts of lytic IgG antibodies and mononuclear cells (156).

Irradiation of tumor cells provides a safe source of antigens for DC uptake. Loading DCs with a mixture of apoptotic and necrotic cells induces phagocytosis and the upregulation of co-stimulatory molecules on DCs (131, 157), leading to improved protective antitumor immunity compared to other loading approaches (158). The presence of the remaining non-phagocytosed apoptotic/necrotic cells is relevant to the process of DC maturation (38, 131), and probably also for the recruitment of host CD207^+^ cells to the vaccination site and draining lymph nodes (39). Recently, radiation-mediated antitumor immunity has been found to require a STING DNA-sensing pathway in DCs, which mediates IFN-β DC production (159). The authors hypothesize that DNA from irradiated tumor cells could be delivered to DCs during cell–cell contact processes. Necrosis can be obtained by repeated freeze-thaw cycles but these lysates assayed as source of tumor antigens have provided discrepant results (157, 160–163). It is likely that their utility is influenced by tumor cell type and whether an immunogenic cell death was induced to generate the lysates. Consequently, and in opposition to the past dichotomy between apoptotic and necrotic tumor cells, approaches would have to be reviewed to evaluate whether the applied procedure renders an immunogenic source of antigens through an immunogenic cell death (164). The enhanced immunogenicity can be mediated by DAMPs being recognized by the PRRs expressed on immune cells (165), or by the presence of chaperone or heat shock proteins (HSPs). The intracellular stimulation of HSPs can be induced by various stressors (166, 167), and HSPs derived from stressed tumor cells are able to bind and transport tumor antigens to APCs. This approach turned out to be an interesting possibility to direct antigens to DCs or as an adjuvant therapy to enhance the immune visibility of poorly immunogenic TAAs (157, 162, 166, 168–170). The isolation method and the type of HSP enriched thereafter are crucial factors that may account for efficacy limitations (171). HSP70-peptide complexes derived from DC-tumor fusion cells are enriched in peptides with superior antigenic properties as compared to its tumor cell counterpart (172). Due to this variability, further experimental investigation is required in this promising field. Finally, DCs are also able to acquire antigens from live cells through a mechanism associated with trogocytosis, where individual DCs physically extract plasma membrane from other cells, generating endocytic vesicles up to 1 μm diameter (173). Along this line, authors assayed a vaccine preparation consisting of a 16-h incubation of DCs with live or apoptotic B16-F10 tumor cells, followed by CD11c^+^ cell enrichment and γ-irradiation before administration (44). Live tumor cells improved cross-presentation by DCs by maintaining antigens in a more native form than apoptotic cells. In a therapeutic assay of B16-F10 lung metastasis, mice were protected upon tumor challenge using the same approach (44, 45). In this study, tumoral protection correlated with lower levels of IL-10 and stronger tumor-specific CD8^+^ T cells response (45).

The origin of the APCs assayed in the vaccine formulation is also relevant. For this reason, allogeneic DC-based vaccines emerged as an alternative strategy to avoid the potential dysfunction of autologous DCs obtained from cancer patients. Allogeneic DCs can induce a stronger vaccine-specific immune response than syngeneic DCs (174). This is thought to trigger a broader T cell reactive repertoire, in which tumor-reactive T cells are generated by incidental cross-reactivity. The enhanced fraction of helper T cells (in response to alloantigens) leads to a better activation of specific CTLs. Stronger NK activity (175, 176), the presence of Th1-type cytokines, and the absence of the Th2-type cytokines IL-10 and IL-4 (177) were also reported. To simulate a clinical trial, Yasuda et al. analyzed the validity of semi-allogeneic DCs, where some MHC class I and II molecules are likely to be shared by recipients. This approach induced the most effective antitumor immune response in a therapeutic setting. While allo-MHC class II molecules may provide favorable T helper activity, it is likely that partial MHC class I matching is required to induce a CTL response. The flawed aspects of this approach are, on one hand, the possible promotion of regulatory T cell expansion, limiting antitumoral immunity via the suppression of not only T helper cells but also CTLs. On the other, repeated vaccination with allogeneic antigens could potentially elicit an alloreaction, which could blunt its immunizing potential (175). Therefore, a more efficient response will probably be provided by syngeneic cancer cells that express allogeneic MHC molecules, which could act as an adjuvant without counteracting the cancer-specific CTL response. Finally, DC culture conditions can introduce variability in relevant aspect as vaccine quality and immunogenic potency. Trivial conditions as detachment (178) and oxygen percentage (179) are able to alter the maturation state of DCs and antigen-specific CTL activation, respectively.

The fusion of DCs and tumor cells results in a heterokaryon without nuclear fusion, which includes molecules from DCs (MHC class I and II, and co-stimulatory molecules) and abundant TAAs that can be efficiently processed and presented by DC-presentation machinery (180). Several animal studies have demonstrated that DCs fused to tumor cells could be administered as a vaccine, eliciting protection upon challenge with tumor cells and regression of established tumors (181–187). Mice immunized with a fusion cell vaccine induced effective cellular and humoral responses against the antigen MUC1 in MUC1-transgenic mice, whose characteristic is to be unresponsive to the MUC1 antigen without a potent stimulation (188). Moreover, they conferred sufficient antitumor immunity to block or delay mammary tumor development in the same model of transgenic mice (189). When translated to human trials, this vaccine strategy might be improved with strategies to inhibit the immunosuppressive activity of Tregs or by combination with more conventional therapies (180). An interesting feature of these cells that is relevant to the clinical setting is that DC-tumor fusion cells could be efficiently frozen without loss of either antigen presentation potency or T-cell stimulatory capacity inducing polyclonal CTL responses (190).

#### Target Receptors for *In Vivo* Antigen Delivery

The development of antigen-coupled APC receptor-specific antibodies, single-chain variable fragments (scFv), and troybodies is another strategy to deliver TAAs to APCs *in vivo*. The ideal target receptor should present a specific pattern of expression to allow the exclusive targeting of APCs. It should be an endocytic receptor, and its activation should result in the presentation of antigen peptides via MHC molecules. Furthermore, in order to induce a potent immunogenic response to the delivered antigen, the process should lead to APC activation and maturation (191). A variety of target molecules have been assayed for mouse antitumor vaccines (MHC class II, CD11c, DEC205, DCIR2, Dectin-1/2, F4/80-like receptor, CIRE, mannose receptor, CD36, Clec 9A, MadCAM, CD80/CD86, CD40, Siglec-H). This strategy requires a lower antigen dosage than uncoupled peptides to stimulate immune responses in mice (42). The main drawback could be the lack of specificity of expression of the targeted molecule. By targeting XCR1, a chemokine receptor exclusively expressed on murine and human cross-presenting DCs, CD8^+^ T cell cytotoxicity could be successfully induced (192). This makes this specific and efficient approach, a very interesting candidate for the clinical setting.

Many of the studies were carried out employing proteins like OVA, BSA, or microbial protein or peptides. Further experimental vaccines using targeted delivery of TAAs may contribute to a better understanding of the potential benefit from this approach. One successful example of translational research is the use of HER2/neu as a target for breast cancer immunotherapy. The delivery of the HER2 antigen targets many DC surface molecules (CD80, CD86, CD11c, CD40, mannose, Fc-γ, and DEC-205), and results in potent immunization with significant CD8^+^ and IFN-γ^+^CD4^+^ T cell responses and cytokine secretion (IFN-γ, TNF-α, and IL-2) (43). More recently, a human trial employing an antibody targeted to DEC-205 fused with the tumor antigen NY-ESO-1 plus TLR agonists as adjuvants demonstrated to be a safe vaccine, effective to mount humoral and cellular response. The inclusion of immune checkpoint inhibitors to overcome the immunosuppressive tumor environment points this design as a promising immunotherapeutic strategy (193).

### Exploring DC response to maturation antigens and vaccine-adjuvant combinations

#### Defining DC Maturation Status

Dentritic cell maturation is a very complex process involving diverse signaling pathways. It is characterized by the acquisition of distinctive functional properties involved in antigen processing and presentation, migration, and T cell co-stimulation (194). Costimulatory DC molecules, MHC-II, and CD40 are usually used to assess DC maturation status and immunostimulating potential. Nonetheless, these do not always correlate with the observed *in vivo* response. Recently, CD70, which binds to CD27 in T cells, has emerged as a very relevant T-cell costimulatory molecule (195, 196). Adoptively transferred CD70-expressing immature DCs were capable of priming CD8^+^ T cells into effectors, to control B16 melanoma tumor growth, to generate complete tumor rejection, and to induce memory CD8^+^ T cells (48). These results highlight the relevance of CD70 readout as an antitumoral efficacy DC marker.

On the other hand, without relying on a few specific markers which may not correlate with *in vivo* DC immunostimulating capacity, DC functionality can be evaluated as a whole by expression profiling. For example, in the steady state, a fraction of cDCs undergo homeostatic (tolerogenic) maturation, upregulating MHC class II molecules to almost the same levels as found under inflammatory conditions (197). Using murine models, it has been possible to isolate different DC subtypes involved in either homeostatic or TLR-induced (immunogenic) maturation, and to characterize the genes involved in these processes (63). Based on bioinformatic analysis, Dalod et al. describe a core set of genes induced in different DC subsets during both homeostatic and TLR-induced maturation (69), suggesting overlapping instructional signals in both maturation processes. On the other hand, there are certain genes that are differentially upregulated (63), and which could account for the difference in DC immunostimulating capacity. Analysis of expression profiles is becoming a useful tool to assess DC functionality, and could eventually lead to predict their immunotherapeutic potential.

#### Potentiating DC Immunotherapeutic Capacity

Tumor necrosis factor α (TNF-α) was one of the first agents assayed that was able to upregulate CD83 in DCs *in vitro*, and thereby improve DC T-cell stimulatory capacity (36). At this time, Jonuleit et al. developed a cytokine cocktail containing the proinflammatory cytokines IL-1β, IL-6, and TNF-α, and prostaglandin E_2_ (PGE_2_) (198), which was used for many years as the gold standard for DC maturation. Although this cocktail efficiently induced the upregulation of DC maturation markers and DC T-cell priming ability, there were impairments observed in IL-12p70 production by human DCs treated with this cocktail (199). IL-12 production is essential in DC-based cancer immunotherapy because of its important role promoting CD8^+^ T cell responses (200). It is tightly regulated, and recently it has been observed that for high expression levels both myeloid differentiation factor 88 (MyD88) and TRIF (TIR-domain-containing adapter-inducing IFN-β)-dependent pathways must be triggered simultaneously (195). Thus, other maturation cocktails are being explored to achieve an optimal IL-12-producing DC maturation.

Using murine models, it was shown that IL-12, as well as type I IFNs, induces a complex gene regulation program, involving chromatin remodeling and the induction of the transcription factors Eomes and T-bet, which are important for Th1 differentiation (201). Many DC-based vaccination studies have explored the use of IL-12 (186, 202). There have been dose-limiting toxicities associated with systemic IL-12p70 administration (203), leading to more careful analysis in preclinical models. The use of IL-12 adjuvancy has now advanced to clinical trials (204–206).

The hematopoietic growth factor and immune modulator, GM-CSF, is another of the most evident DC-based vaccine adjuvants, as it was the first cytokine described to efficiently promote DC development *in vitro* (207). GM-CSF has been shown to increase antitumoral effects when administered or produced locally at the vaccination site (208–210). Both GM-CSF and Flt3L are capable of inducing local and systemic expansion of DCs when used as adjuvants, but the antitumoral efficacy of GM-CSF is significantly higher (211).

The complex combination of TLR expression in different murine DC subsets and monocytes is essential for their functional specialization (63, 69). TLR triggering in DCs induces NF-kβ activation (212) and subsequently, the production of pro-inflammatory cytokines that are important not only for the innate immune response but also for T-cell polarization. TLR3, TLR7, TLR8, and TLR9 are intracellular TLRs recognizing nucleic acids. Nucleic-acid sensing TLRs and TLR4 are able to induce IRF3 or IRF7 activation, leading to type I IFN expression. Type I IFNs can induce Th1 differentiation and IFN-γ production (213), and thus cytotoxic CD8^+^ T cell responses. In particular, IFN-β signaling in CD8^+^ DCs has been found to be responsible for spontaneous tumor antigen-specific T cell priming and tumor rejection (105). Triggering TLR3, TLR7, and TLR9 can also enhance antigen cross-presentation by DCs (79, 214). Thus, triggering these nucleic-acid sensing TLRs in DCs is a good strategy to elicit potent antitumor responses, and several combinations and administration schedules are being evaluated.

A synergistic effect was observed when activating both TLR7 and TLR3 in GM-CSF-cultured bone-marrow derived DCs (215). DCs stimulated with R-848 (TLR7/8 agonist) and Poly I:C (TLR3 agonist) were stronger stimulators of specific CD8^+^ and CD4^+^ T cells *in vitro* and induced superior CTL priming *in vivo*. This was probably due to the simultaneous activation of the MyD88-independent pathway triggered by TLR3 and the MyD88-dependent pathway triggered by TLR7. In another work, Flt3-cultured bone marrow-derived DCs were also stimulated with various concentrations of several TLR agonists (37). Simultaneous stimulation of TLR4, which signals through MyD88 and/or TRIF, and TLR2 or TLR7, which signal through MyD88-dependent pathways, led to higher levels of IL-6 and IL12p70 production. Interestingly, they also observed a synergistically enhanced production of inflammatory cytokines when triggering TLR7 with TLR2 or TLR9, all of which signal through MyD88-dependent signaling pathways. Thus, it is important in order to maximize DC activation, to explore triggering simultaneously different TLRs and signaling pathways.

Due to their DC-stimulating capacity, TLR agonists have been assayed as adjuvants for many cancer immunotherapies. Above all, TLR7 agonists are especially interesting candidates because apart from activating APCs, they induce T-cell, NK, and NKT activation *in vitro* (46). Oral doses of the TLR7 agonist imiquimod generate antitumoral responses in several murine tumor models as MC-26 colon carcinoma, LLC, and RIF-1 sarcoma (216). Additionally, the combination of DC-based immunotherapies with peritumoral or topical imiquimod has also shown to be a successful approach (46, 217). Topical administration of imiquimod cream induces a strong inflammatory response in the skin and enhances migration of LCs or immature GM-CSF/IL-4-cultured bone marrow-derived DCs to the draining lymph nodes of treated mice (40, 41), inducing a potent CTL response (41). More recent works show that imiquimod skin treatment leads to local recruitment of pDCs and induces melanoma tumor regression (78). It was demonstrated that pDCs are required for the antitumoral imiquimod-mediated effect. Furthermore, the s.c. administration of a GM-CSF gene-transduced tumor vaccine (GVAX) and imiquimod induced the recruitment of activated pDCs to tumor vaccine sites and tumor draining lymph nodes, and elicited the suppression of tumor growth (210).

Sanchez et al. proved *in vivo* that OVA immunization with a combination of TLR and CD40 agonists generated more potent primary and memory CD8^+^ T cell responses that either agonist alone (49). They report a synergistic effect when combinations of TLR and CD40 agonists were administered i.p. together. They argue that the increase in classical DC activation markers, such as CD80, CD86, and CD40 induced by TLR agonists is necessary for the initial CD28-dependent CD8^+^ T cell stimulation, but that CD70 induction by anti-CD40 is needed to stimulate long-term memory. Furthermore, CD70 binding to CD27 in T cells was found to be critical for potent CD8^+^ T cell responses, since antigen-specific CD8^+^ T cell expansion *in vivo* was abrogated by CD70 blockage. The authors observed that the combined stimulation of TLR and CD40 pathways gives maximal CD70 expression. In a promising clinical trial for advanced melanoma, autologous DCs were electroporated with mRNA encoding CD40L, a constitutively active TLR4 and CD70 (TriMix), thereby improving DCs immunostimulatory capacity (218). In another melanoma clinical trial, autologous DCs were activated with CD40L and IFN-y, and a higher level of IL-12p70 production by patient’s DCs correlated with a better clinical outcome. Interestingly, IL-12p35 deficient production by some patient’s DCs could be corrected *in vitro* by using TLR agonists like poly I:C and R848.

Other TLR-independent innate signaling pathways were found to cooperate with the adaptive signaling CD40 pathway to induce CD70 expression in DCs and a potent CD8^+^ T cell response *in vivo* (50). These TLR-independent stimuli include type I IFNs and α-GalCer (219, 220). Maximal CD70 expression and CD8^+^ T cell memory were found to be induced when α-GalCer or type I IFNs were used in combination with anti-CD40 antibody (50). Therefore, CD40 triggering in combination with TLR agonists or TLR-independent stimuli like type I IFN and α-GalCer is a promising strategy for inducing DC maturation in cancer immunotherapy approaches.

In cancer, vaccine-induced immunity may be dampened by self-regulatory mechanisms, and adjuvants may exacerbate them. Indeed, interesting experiments in a mammary carcinoma murine model have shown that when imiquimod was administered intradermally with the self-antigen IGFBP-2, no antitumor effect was elicited due to the induction of a potent immunosuppressive regulatory response (221). Serum levels of IL-10 and systemic levels of myeloid-derived suppressor cells and Tregs were increased. Furthermore, when GM-CSF was applied as a sole adjuvant, it significantly inhibited tumor growth, but when combined i.d. with imiquimod, the antitumoral effect was abrogated. On the other hand, when imiquimod was used by the same authors in an exogenous OVA-peptide based immunization, DC maturation and mobilization, and OVA-specific CD8^+^ and CD4^+^ T cells were detected. This emphasizes the importance of extensive preclinical modeling in cancer vaccine development, and the need of models that do not rely on exogenous antigens in order to detect self-regulatory mechanisms.

## Conclusion

Dendritic cell-based vaccination has the potential to make a difference in cancer treatment. It is not sufficient to be a safe approach and to elicit measurable immunological responses. A potent CD8^+^ T cell effector memory response should be triggered, able to control and eliminate tumors. The rationale to design these vaccines comes from clinical observations and, as depicted in this work, from pertinent investigations performed using murine models. However, each model has specific limitations and biases. Established cell lines, which are easily propagated and studied, transplanted into syngeneic mice have been utilized for many decades. Although they have answered many questions regarding DC functionality and DC use in cancer vaccines, their contribution to approved therapeutics has been limited. This could be due to the fact that heterogeneity and diversity of human cancers are not covered. Though more technically challenging, GEM models, which recapitulate genetic alterations present in human tumors, and humanized mice, which allow the study of human tumors in the context of their own immune system, are useful tools that will contribute to the design of more effective DC-based vaccines.

It is of great importance in order to break immune tolerance to the tumor to induce a proper activation of DCs by triggering several activation pathways. As discussed in this work, this can be done by *ex vivo* manipulation of DCs, or by using the appropriate adjuvants to boost DC response *in vivo*. The selection of adjuvant and site of administration will result in the activation of a distinct APC profile. Furthermore, given that synergy and cooperation between DC subsets has been observed, a vaccine design that targets several DC population presents itself as a potent immunization strategy. As shown in this work, DC-based therapies have been efficient in activating tumor-specific CD8^+^ T cells. In patients, analysis of the tumoral microenvironment has shown T-cell infiltration (222), thus immune failure appears to occur in the effector phase, with a dominant effect of inhibitory mechanisms within the tumor. As has been observed in a recent clinical trial (193), DC-based immunotherapies will surely benefit from combination with recently developed immunomodulatory agents that block negative regulators of T cell immunity, opening a promising field in cancer immunotherapy.

The results presented in this work show that many authors report *in vitro* immunological and short-term protection results using murine models. We conclude that this is not sufficient to draw relevant conclusions involving the efficacy of DC-based therapies. It is important to perform experiments analyzing long-term tumoral protection and memory CD8^+^ T-cell profile, and identifying the tumoral antigens that are able to generate long-lasting responses. To help the field move forward, it is vital to reach a consensus in this matter.

## Conflict of Interest Statement

The authors declare that the research was conducted in the absence of any commercial or financial relationships that could be construed as a potential conflict of interest.
